# Functional Characterization of the *EMBRYONIC FLOWER 2* Gene Involved in Flowering in *Ginkgo biloba*

**DOI:** 10.3389/fpls.2021.681166

**Published:** 2021-06-21

**Authors:** Xian Zhou, Lanlan Wang, Janping Yan, Jiabao Ye, Shuiyuan Cheng, Feng Xu, Guiyuan Wang, Weiwei Zhang, Yongling Liao, Xiaomeng Liu

**Affiliations:** ^1^College of Horticulture and Gardening, Yangtze University, Jingzhou, China; ^2^National R&D for Se-rich Agricultural Products Processing Technology, Wuhan Polytechnic University, Wuhan, China

**Keywords:** *Ginkgo biloba*, *GbEMF2*, flowering, *emf2* mutant, RNA-seq

## Abstract

*Ginkgo biloba* has edible, medicinal, and ornamental value. However, the long juvenile phase prevents the development of the *G. biloba* industry, and there are few reports on the identification and functional analysis of genes regulating the flowering time of *G. biloba*. EMBRYONIC FLOWER 2 (EMF), an important protein in flower development, functions to promote vegetative growth and repress flowering. In this study, a novel *EMF* gene (*GbEMF2*) was cloned and characterized from *G. biloba*. *GbEMF2* contains a 2,193 bp open reading frame (ORF) encoding 730 amino acids. GbEMF2 harbors conserved VEFS-Box domain by the plant EMF protein. The phylogenic analysis showed that GbEMF2 originated from a polycomb-group (Pc-G) protein ancestor and was a member of the EMF2 protein. The quantitative real-time PCR (qRT-PCR) analysis revealed that *GbEMF2* was expressed in all detected organs, and it showed a significantly higher level in ovulating strobilus and microstrobilus than in other organs. Compared with *emf2* mutant plants, overexpression of *GbEMF2* driven by the CaMV 35S promoter in *emf2* mutant *Arabidopsis* plants delayed flowering but earlier than wild-type (WT) plants. This result indicated that *GbEMF2* repressed flowering in *G. biloba*. Moreover, the RNA-seq analysis of *GbEMF2* transgenic *Arabidopsis* plants (*GbEMF2-OE/emf2*), WT plants, and *emf2* mutants screened out 227 differentially expressed genes (DEGs). Among these DEGs, *FLC*, *MAF5*, and *MAF5-1* genes were related to flower organ development and regulated by *GbEMF2*. In addition, some genes participating in sugar metabolism, such as *Alpha-amylase 1* (*AMY1*), *BAM1*, and *Sucrose synthase 3* (*SUS3*) genes, were also controlled by *GbEMF2*. Overall, our results suggested that *GbEMF2* negatively regulates flowering development in *G. biloba*. This finding provided a foundation and target gene for shortening the Ginkgo juvenile period by genetic engineering technology.

## Introduction

*Ginkgo biloba* L. (*G. biloba*), which has a long life and is known as “gongsun tree,” is only one of the Ginkgoaceae and appeared in the Mesozoic era with an extremely long juvenile stage (Ye et al., 2019). *Ginkgo biloba* has a long history of cultivation in China and is widely used in the fields of landscaping, medicine, and food (Crane, 2018). It usually takes 15–20 years for Ginkgo trees to blossom and bear fruit, which is a serious obstacle to breed good Ginkgo varieties (Yan et al., 2017). For many years, breeding scientists have tried to shorten the infancy of *G. biloba* through asexual propagation methods, such as cutting and grafting. Though in that case, blossoming and bearing fruit takes 6–8 years, it is still a long breeding cycle for trees. Thus, new approaches are needed to further shorten the infancy of *G. biloba*. Shortening the time from infancy to flowering mechanism in study of *Arabidopsis thaliana* (Yoshida et al., 2001), *Glycine max* (Cai et al., 2018), and apple (Charrier et al., 2019) maybe provide a reference for *G. biloba*.

Flowering, a key developmental trait, has attracted much attention from researchers. With the in-depth study of plant flowering in molecular biology, the theory for a flowering regulation model has been gradually deepened, from the most classic ABC model (Coen et al., 1990) to the classic “Enabling, Promoting, Resetting” model proposed by Boss et al. (2004). Numerous flowering genes are involved in these model systems by regulating different flowering pathways in plants. The development of woody plants is comprised of main stages from vegetative growth to flowering. The first stage is the transition from vegetative growth to reproductive growth, which is synergistically regulated by many genes. Some of them are transcriptional regulators, such as *FLOWERING LOCUS C* (*FLC*; Wu et al., 2016), *CONSTANS* (*CO*; Mulki and von Korff, 2016), and *EMBRYONIC FLOWER 2* (*EMF*; Liu et al., 2012). Some genes function as signal transduction, for instance, *FLOWERING LOCUS T* (*FT*; Navarro et al., 2011), *TERMINAL FLOWER* (*TFL*; Wickland and Hanzawa, 2015). The second stage is the transition from inflorescence to flowering. In this stage, the genes associating with flowering mainly include meristem-specific genes and flower organ-specific genes (MADS-box family), which regulate the formation of meristems and flower organs in time and space. For example, LEAFY (*LFY*) and *APETALA1* (*AP1*) play important roles in flowering initiation in the meristem (Weigel et al., 1992; Eckardt, 2006). The development of flower organs depends on many flower organ-specific genes, such as *AGAMOUS*, *AP1*, *AP3*, and *PISTILLATA* (*PI*; Krizek and Fletcher, 2005). Overexpressing and silencing the *MAF1* gene led to late flowering and early flowering, respectively. In addition, *MAF1* directly repressed *AP3* and activated *MAF2*, which negatively regulated flowering (Huang et al., 2018). *FRIGIDA* (*FRI*) delayed flowering by activating the expression of target genes *FLC*, *MAF4*, and *MAF5* (Kong et al., 2019). *FLC*, acting as a MADS-box transcription factor and a floral repressor, regulates flowering by directly repressing downstream genes, such as *FT* and *SUPPRESSOR OF OVEREXPRESSION OF CONSTANS1* (*SOC1*; Deng et al., 2011). This regulating process was enhanced by the interaction of *DELLA* and *FLC* (Li et al., 2016a). Further, DELLAs interacted with *SQUAMOSA PROMOTER BINDING PROTEIN-LIKE* (*SPL*) to interfere *SPL* transcriptional activity and affect flowering time (Yu et al., 2012). At present, some flowering-related genes have been identified in *G. biloba*, such as *GbCO* (Yan et al., 2017), *GbFT* (Wang et al., 2019), *GbMADS2* (Wang et al., 2015), *GbSEP* (Cheng et al., 2016), and *GbMADS9* (Yang et al., 2016).

The *EMF*, an important gene inhabiting the flowering of plants, has become a hot topic in recent years. Thus far, scientists have cloned *EMF* gene family members in *A. thaliana* (Yoshida et al., 2001), rice (Li et al., 2006), broccoli (Liu et al., 2012), bamboo (Xu et al., 2010), and cotton (Ma et al., 2020). The members of the *EMF* gene family were *EMF1* and *EMF2*, which encode proteins with great differences in sequence and structure. EMF2, FERTILIZATION INDEPENDENT SEED2 (FIS), and VERNALIZATION2 (VRN2) encode Suppressor of zeste 12 [Su(Z)12] subunit, and EMF2, FIS2, and VRN2 are homologous proteins of Su(Z)12 (Chaudhury et al., 1997; Gendall et al., 2001; Yoshida et al., 2001). Studies in model plants, such as *A. thaliana*, have shown that EMF regulates the differentiation of plant vegetative growth to the flower meristem, and its expression determines the time of plant vegetative growth (Sung et al., 1992; Yang et al., 1995) and is closely related to the development of inflorescence organs (Chen et al., 1997; Chanvivattana et al., 2004). *EMF1* and *EMF2* are constitutively expressed in the roots, rosette leaves, stem, mature leaves, and other vegetative organs and flower clusters of *A. thaliana* (Aubert et al., 2001; Yoshida et al., 2001). However, these findings are based on the research of annual herbs, and which viewpoint can correctly reflect the expression pattern of *EMF* genes in the woody plant development that remains to be further explored. Therefore, *GbEMF2*, one member of the *EMF* gene family, was cloned and characterized from *G. biloba*, a gymnospermous tree, in this study. *GbEMF2* was transferred into *Arabidopsis emf2* mutants, which verified that *GbEMF2* was also involved in the regulation of flowering. Our findings not only establish a foundation for elucidating the gene regulation network of Ginkgo flowering, but also provide a target gene for using the genetic engineering technology to shorten the infancy of woody plants.

## Materials and Methods

### Plant Materials and Growth Conditions

Plant materials were collected from 31-year-old *G. biloba* “Jiafoshou” tree grown in the Ginkgo Science and Technology Garden, Yangtze University (around N30.35, E112.14). The roots, stems, leaves, microstrobilus, ovulate strobilus, and immature fruits of Ginkgo grafts were collected to test the spatial expression profile of *GbEMF2*. The harvested samples were rapidly frozen in liquid nitrogen and stored at −80°C in reserve.

*Arabidopsis* wild-type (WT) Landsberg erecta seeds and *emf2* mutant seeds (SALK_115527) were purchased from the Arabidopsis Biological Research Center of Ohio State University, Columbus, OH, United States. The mutant seeds of *emf2* were all grown in the MS plates with 0, 50, or 100 mg/L kanamycin, cold stratified at 4°C for 72 h, then transferred to an artificial climate incubator for germination and development. The artificial climate incubator was set to 16 h of light, 25°C, 8 h of darkness, at 18°C, 12,000 lex of light, and 70% humidity. About 15-day-old seedlings were transferred into soil and grown under long-day conditions for the observation of bolting and flowering development.

### Cloning of Full-Length *GbEMF2* cDNA

Total RNA was extracted from *G. biloba* microstrobilus using a TaKaRa MiniBEST Plant RNA Extraction Kit (Takara Bio Inc., Dalian, China). The first strand of cDNA was synthesized using the PrimeScript™ 1st cDNA Synthesis Kit. Specific primers (GbEMF2-F and GbEMF2-R; Takara Bio Inc., Dalian, China; Supplementary Table S1) were designed based on the EMF2 unigene sequence (CL9451Contig1) from *G. biloba* transcriptome data. The *GbEMF2* gene was amplified using PCR under the following program: 94°C for 3 min; 33 cycles of 94°C for 30 s, 55°C for 30 s, 72°C for 90 s; and a final extension at 72°C for 10 min. The PCR product was tested with 1% agarose gel electrophoresis, and the target fragment was recovered by an Agarose Gel DNA Purification Kit (TaKaRa, Dalian, China). Then, the target fragment was ligated into the pMD19-T vector and transformed into DH5α. The single colony was picked and cultured. Screened positive clones were sent to Shanghai Sangon Biotech (Shanghai, China) for sequencing.

### Bioinformatics and Molecular Evolution Analyses

Primers were designed using the Primer 5 software and the online PrimerQuest Tool.^1^ Higher similarity sequences were obtained by using the BLAST tool^2^ for homologous alignments. The DNA sequence analysis was completed by using the Vector NT I 11.5 (Invitrogen) and DNAMAN software.^3^ The protein sequence and oter homologous sequences on GenBank were analyzed using the CLUSTAL X2^4^ and MEGA6 software,^5^ and a phylogenetic tree was constructed by the Neighbor-Joining method.

### Quantitative Real-Time PCR Analysis

Total RNA was extracted from six organs of *G. biloba* using a TaKaRa MiniBEST Plant RNA Extraction Kit (TaKaRa, Dalian, China). The total RNA was isolated from each sample by using the PrimeScript™ RT Reagent Kit with the gDNA Eraser (Perfect Real Time; TaKaRa, Dalian, China). First-strand cDNA was generated from 1 μg of total RNA. cDNA was diluted 10 times as a template. The primers, GbEMF2-dF and GbEMF2-dR (Supplementary Table S1), were designed for the quantitative real-time PCR (qRT-PCR) amplification. *GbGAPDH* (Meng et al., 2018) was used as the internal reference gene, and its primers were GbGAPDH-F and GbGAPDH-R (Supplementary Table S1). qRT-PCR was performed using the BioEasy Master Mix (SYBR Green Mix BIOER, Hangzhou, China) according to the instructions of the manufacturer. The reaction system was 20 μl and contained the following: 10 μl of 2 × SYBR Green Mix, 0.2 μl of each primer (10 μM), 2 μl of diluted cDNA, and 7.6 μl of nuclease-free water. The PCR program was as follows: stage one, 95°C for 30 s; stage two, 40 cycles of 95°C for 10 s and 60°C for 30 s; stage three, 95°C for 15 s, 60°C for 1 min, and 95°C for 15 s. Three biological replicates were prepared per sample, and relative expression levels calculations, which were performed using the 2^-△△Ct^ method (Schmittgen and Livak, 2008).

### Vector Construction and *Arabidopsis* Transformation

The *GbEMF2* was amplified using GbEMF2-X and GbEMF2-B (Supplementary Table S1). The restriction enzyme cutting site of *Xba* I and *Bam* HI was introduced into GbEMF2-X and GbEMF2-B, respectively. The pBI121-GUS vector was rebuilt and saved by our laboratory. The recombinant GbEMF2-pBI121-GUS vector was obtained by inserting the open reading frames (ORFs) of *GbEMF2* into the pBI121-GUS vector digested with Xba I and Bam HI with T4 DNA ligase (TaKaRa, Dalian, China). Recombinant GbEMF2-pBI121-GUS vector was introduced into Agrobacterium tumefaciens strain LBA4404 by the liquid nitrogen freezing–thawing method for *A. thaliana* transformation of *emf2* mutant using the floral dip method (Clough and Bent, 1998).

### Screening and Detection of Transgenic *A. thaliana*

Putative transformants were selected on the MS plates with 100 mg/L kanamycin (primary transformants were defined as T_0_). T_0_ seeds were sown on the MS plates with 100 mg/L kanamycin and cold stratified at 4°C for 72 h. Later, the seeds were transferred to an artificial climate incubator for germination and development. The artificial climate incubator was set to 16 h of light, 25°C, 8 h of darkness, 18°C, 12,000 lex of light, and 70% humidity. About 15-day-old seedlings were transferred into soil and grown under long-day condition. T_1_ and T_2_ transgenic plants were further confirmed by PCR and β-glucuronidase activity, referring to the method of Wang et al. (2019). T_3_ seeds from T_2_ transgenic plants were harvested following the screening by the MS plates with 100 mg/L kanamycin. The flowering time of T_3_ *GbEMF2* transgenic plants, *emf2* mutants, and WT *A. thaliana* plants was recorded (Hanano and Goto, 2011).

### RNA-Seq Analysis and qRT-PCR Validation

The WT, *emf2* mutants, and *GbEMF2-OE/emf2* (T_3_ generation) transgenic of *A. thaliana* plants were planted in an artificial climate incubator. The samples (selected above-ground part of the plants) were collected when the *GbEMF2-OE/emf2* transgenic was blooming and sent to Biomarker Biotechnology Corporation (Beijing, China) for RNA-seq. Every sample had three biological replicates, and one replicate had 10 plants. Raw data were generated by the Illumina HiSeq 2500 High-Throughput Sequencing (Illumina, San Diego, CA, United States). After removing low-quality clean reads, high-quality clean reads were aligned to the genome data of *A. thaliana* by HISAT2 (Kim et al., 2015) and assembled by StringTie^6^ (Pertea et al., 2015). The download for genome data of *A. thaliana* is available at https://www.arabidopsis.org/download/index-auto.jsp?dir=%2Fdownload_files%2FGenes%2FTAIR10_genome_release%2FTAIR10_chromosome_files. To compare the *GbEMF2* T_3_ group with the *emf2* mutant and WT groups, the differentially expressed genes (DEGs) were annotated with Nr^7^ (NCBI nonredundant protein sequences), Swiss-Prot^8^ (a manually annotated and reviewed protein sequence database), gene ontology (GO) annotation,^9^ COG annotation,^10^ KOG,^11^ Protein family (Pfam),^12^ and Kyoto Encyclopedia of Genes and Genomes (KEGG).^13^ We screened for gene differential expression by DESeq2. Genes with a fold change of ≥2 and FDR < 0.01 were defined as DEGs. qRT-PCR with random selection, some DEGs were conducted for verifying RNA-seq data. *AtActin* was selected as the internal reference gene of *A. thaliana*, and its primers were AtActin-F and AtActin-R (Supplementary Table S1). RNA samples were returned by Biomarker Biotechnology Corporation (Beijing, China) for validation of RNA-seq. Three biological replicates were prepared per sample, and relative expression levels calculations, which were performed using the 2^-△△Ct^ method (Schmittgen and Livak, 2008). qRT-PCR was performed using the BioEasy Master Mix (SYBR Green Mix; BIOER, Hangzhou, China) according to the instructions of the manufacturer. The reaction system was 20 μl and contained the following: 10 μl of 2 × SYBR Green Mix, 0.2 μl of each primer (10 μM), 2 μl of diluted cDNA, and 7.6 μl of nuclease-free water. The PCR program was as follows: stage one, 95°C for 30 s; stage two, 40 cycles of 95°C for 10 s and 60°C for 30 s; stage three, 95°C for 15 s, 60°C for 1 min, and 95°C for 15 s. The qRT-PCR primers of selected DEGs are shown in Supplementary Table S1.

## Results

### Cloning and Characterization of *GbEMF2* in *G. biloba*

**Figure 1:**
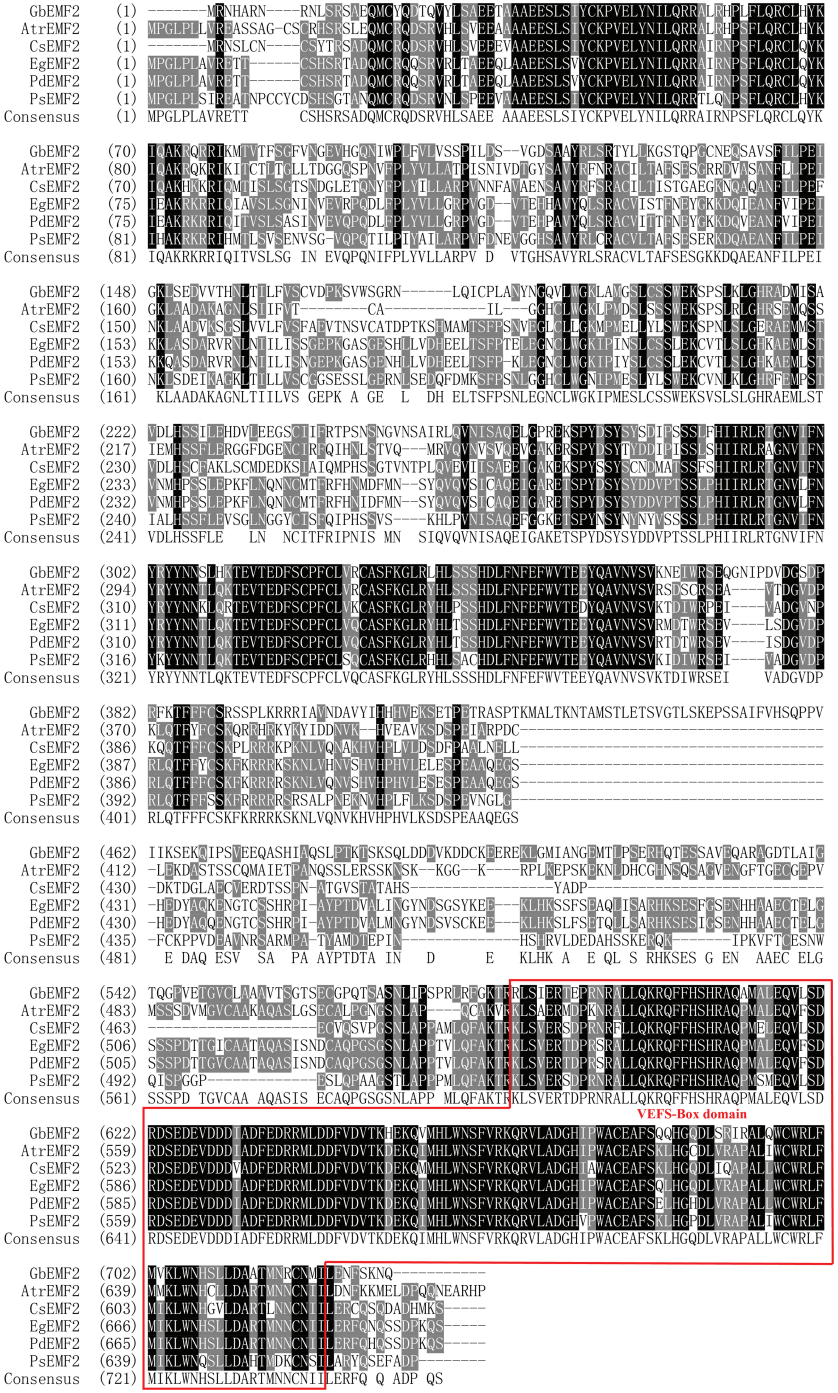
Similarity analysis of GbEMF2 protein and EMF2 proteins in other species. GbEMF2 (*Ginkgo biloba*), AtrEMF2 (*Amborella trichopoda*), EgEMF2 (*Elaeis guineensis*), PdEMF2 (*Phoenix dactylifera*), PsEMF2 (*Papaver somniferum*), and CsEMF2 (*Camellia sinensis*). Shaded in black are identical sequence. Shaded in gray are conservative sequences. The red box is VEFS-Box conserved domain of EMF2 homologous protein family.

**Figure 2:**
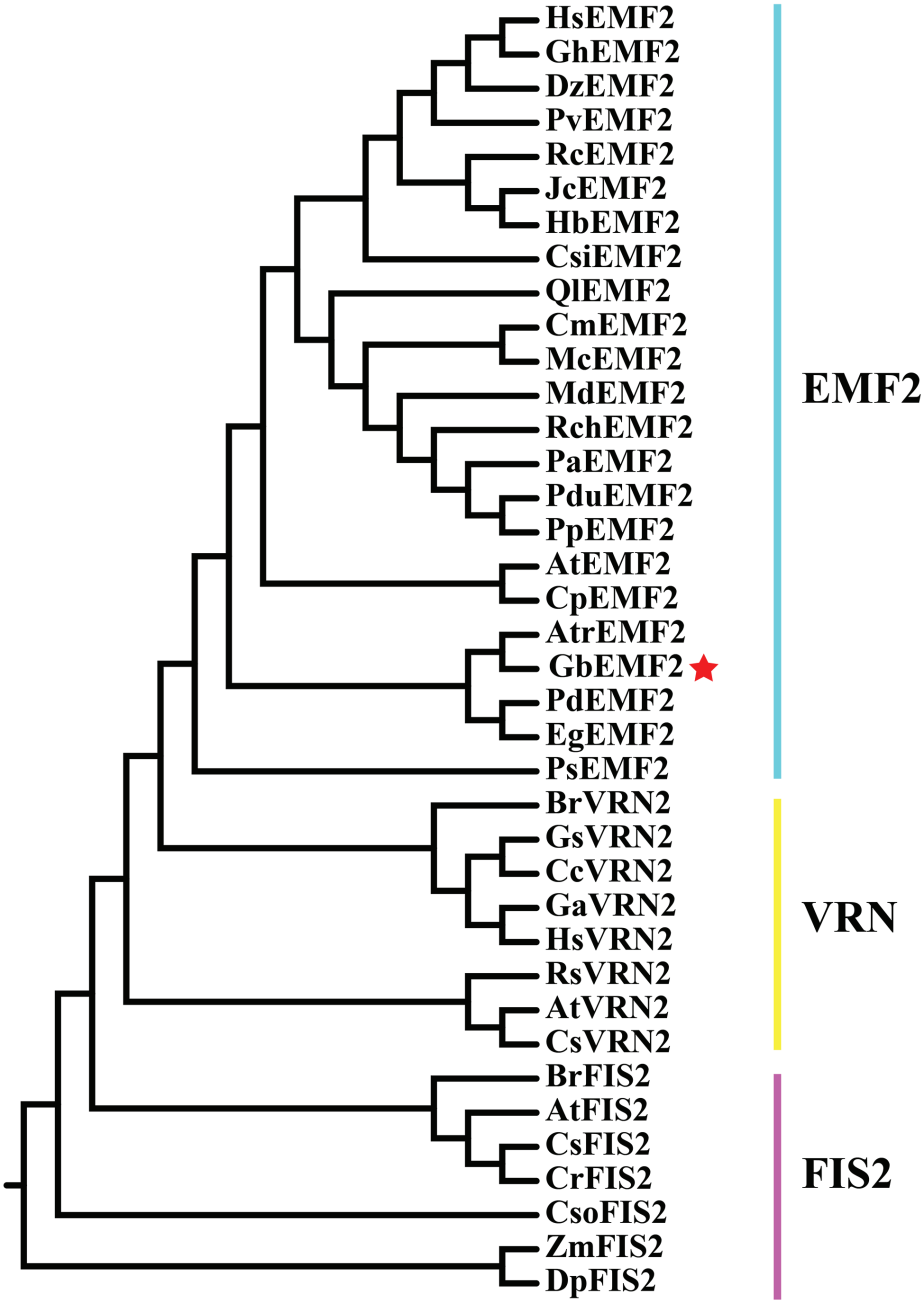
Phylogenetic tree of protein in Su(Z)12 homologous protein family.

The *GbEMF2* gene was cloned from *G. biloba* (GenBank accession no. MH791443) and contained a 2,193 bp ORF, which encodes a 730 amino acid protein (Supplementary Figure S1). Its theoretical molecular weight and pI were 82.6 kDa and 7.33, respectively. The deduced amino acid sequence of GbEMF2 has 55, 54, 55, 52, and 54% similarity with AtrEMF2 (*Amborella trichopoda*), EgEMF2 (*Elaeis guineensis*), PdEMF2 (*Phoenix dactylifera*), PsEMF2 (*Papaver somniferum*), and CsEMF2 (*Camellia sinensis*), respectively (Supplementary Table S2). Furthermore, the alignment analysis of these amino acid sequences showed the homology of GbEMF2 with EMF2 proteins of the amborellales (*Amborella trichopoda*), dicotyledons (*P. dactylifera*, *P, somniferum*, and *C. sinensis*) and monocotyledon (*E. guineensis*; Figure 1). The conserved domain analysis demonstrated that GbEMF2 contained the VEFS-Box domain belong to a member of Su(Z)12. We constructed a phylogenetic tree for understanding the evolution of GbEMF2 (Supplementary Table S3). As the phylogenetic tree is shown (Figure 2), EMF2 protein, VRN2 protein, and FIS2 protein originated from a common ancestor. GbEMF2 was divided into EMF2 protein and had the closest relationship with AtrEMF2, EgEMF2, PdEMF2, VvEMF2, and RcEMF2.

### Expression Patterns of *GbEMF2* in Different Organs of *G. biloba*

**Figure 3:**
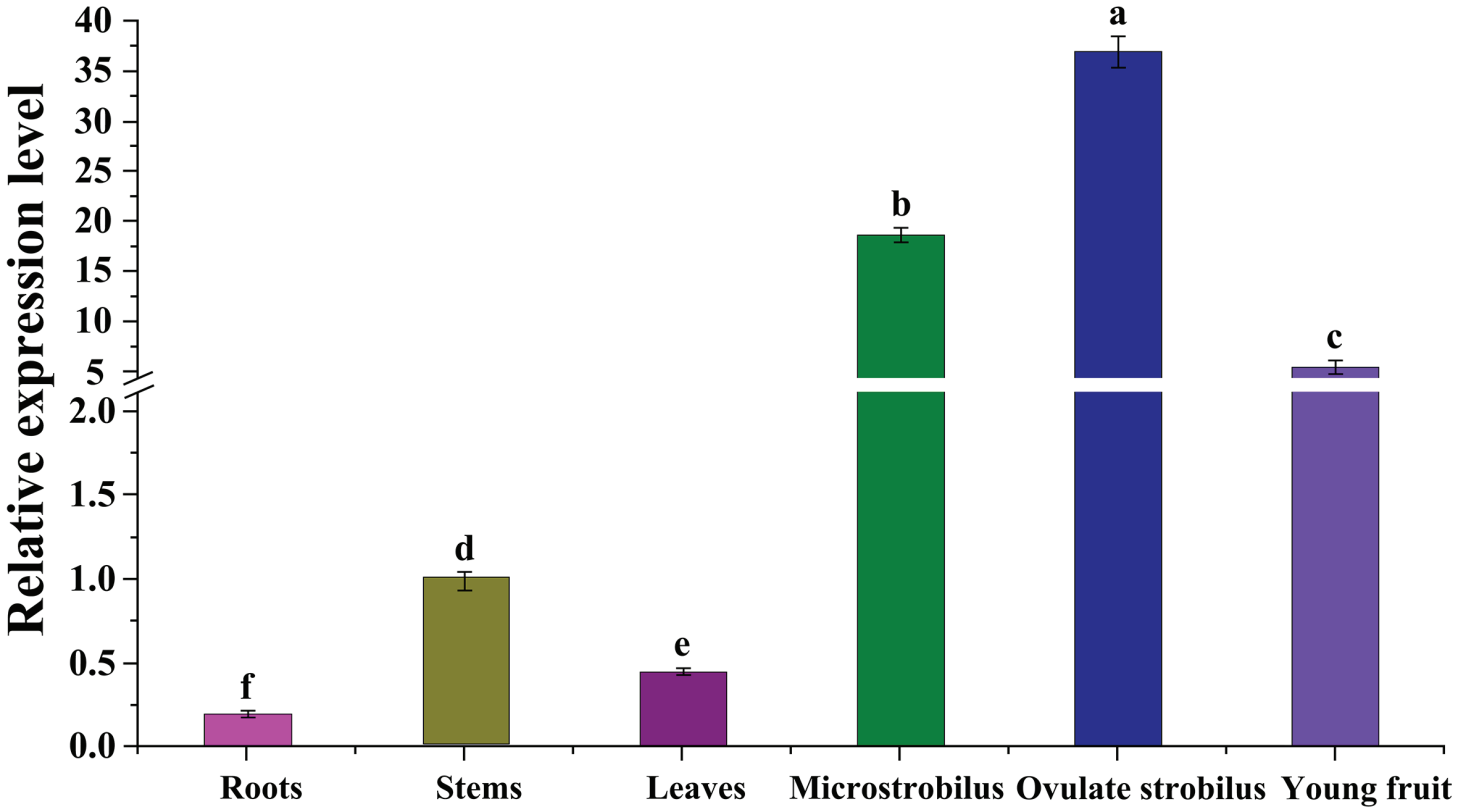
Expression patterns of *GbEMF2* in different organs of *G. biloba* with quantitative real-time PCR (qRT-PCR). Data are means ± SD for *n* = 3 biological replicates. Letters mean significant difference at *p* ≤ 0.05 by the Duncan’s multiple range tests.

To characterize the function of *GbEMF2*, we performed qRT-PCR experiments in different Ginkgo organs. Our results showed that *GbEMF2* was detected in the measured organs (Figure 3). The expression level of the reproductive organs was significantly higher than that of the vegetative organs. In particular, the *GbEMF2* gene was mainly expressed in ovulate strobilus and microstrobilus with a significantly higher level than that in immature fruits, stems, leaves, and roots. The expression level of *GbEMF2* was rarely observed in the roots.

### Ectopic Overexpression of *GbEMF2* in *Arabidopsis* Plants

**Figure 4:**
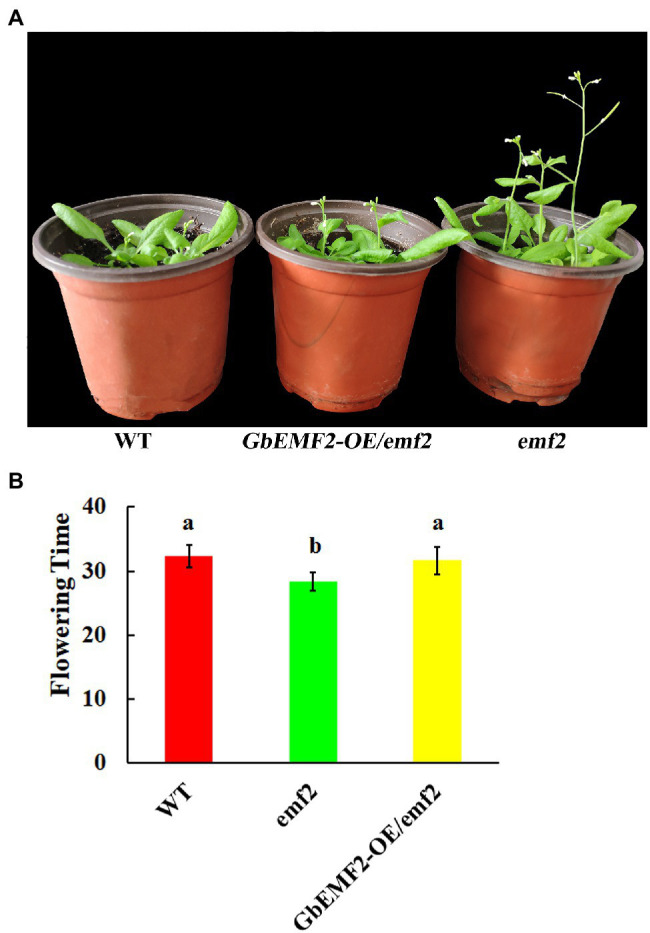
Comparative analysis of the flowering time in *GbEMF2-OE/emf2*, wild-type (WT), and *emf2* mutant of *Arabidopsis*. *GbEMF2-OE/emf2*, T_3_ generation of GbEMF2 transgenic *Arabidopsis*; WT, wild-type *Arabidopsis*; *emf2*, *Arabidopsis emf2* mutant plants. **(A)** Comparison of morphological characteristics of the flowering time. **(B)** Comparison of the days of flowering time of different plants. Each sample has three biological replicates, and data are mean ± SD for three biological replicates, with 10 plants per replicate. Letters mean significant difference at *p* ≤ 0.05 by the Duncan’s multiple range tests.

To further investigate the function of *GbEMF2* in flowering, *GbEMF2* was transformed into *Arabidopsis* plants for heterologous expression. After harvesting T_0_ seeds from primary transformants of *A. thaliana*, the MS medium containing 100 mg/L kanamycin was used to screen GbEMF2 transgenic plants (Supplementary Figure S2). The T_2_ generation plants were screened with kanamycin (Supplementary Figure S3A). To further determine whether *GbEMF2* had been successfully transformed into *A. thaliana*, the T_2_ generation plants were also tested by GUS staining (Supplementary Figure S3B) and the PCR verification with DNA which was served as template (Supplementary Figures S3C,D). After obtaining the verified T_3_ generation of transgenic *A. thaliana* (*GbEMF2-OE*/*emf2*), WT, and *emf2* mutants were planted for phenotypic comparative observation and flowering time. As shown in Figures 4A,B, *emf2* mutant flowered significantly earlier than the WT and *GbEMF2-OE*/*emf2*. The *emf2* mutant bloomed 28 days after sowing, whereas *GbEMF2-OE*/*emf2* flowered approximately 31 days and WT flowered around 32 days after sowing, respectively. Taken together, our data showed that the overexpression of the *GbEMF2* gene restored the phenotype of premature flowering in *Arabidopsis emf2* mutant plants. It is indicated that the *GbEMF2* gene might regulate flowering in *G. biloba*.

### Identification of DEGs Among the WT, *emf2* Mutants, and *GbEMF2* Transgenic *Arabidopsis* Plants by RNA-Seq Analysis

**Figure 5:**
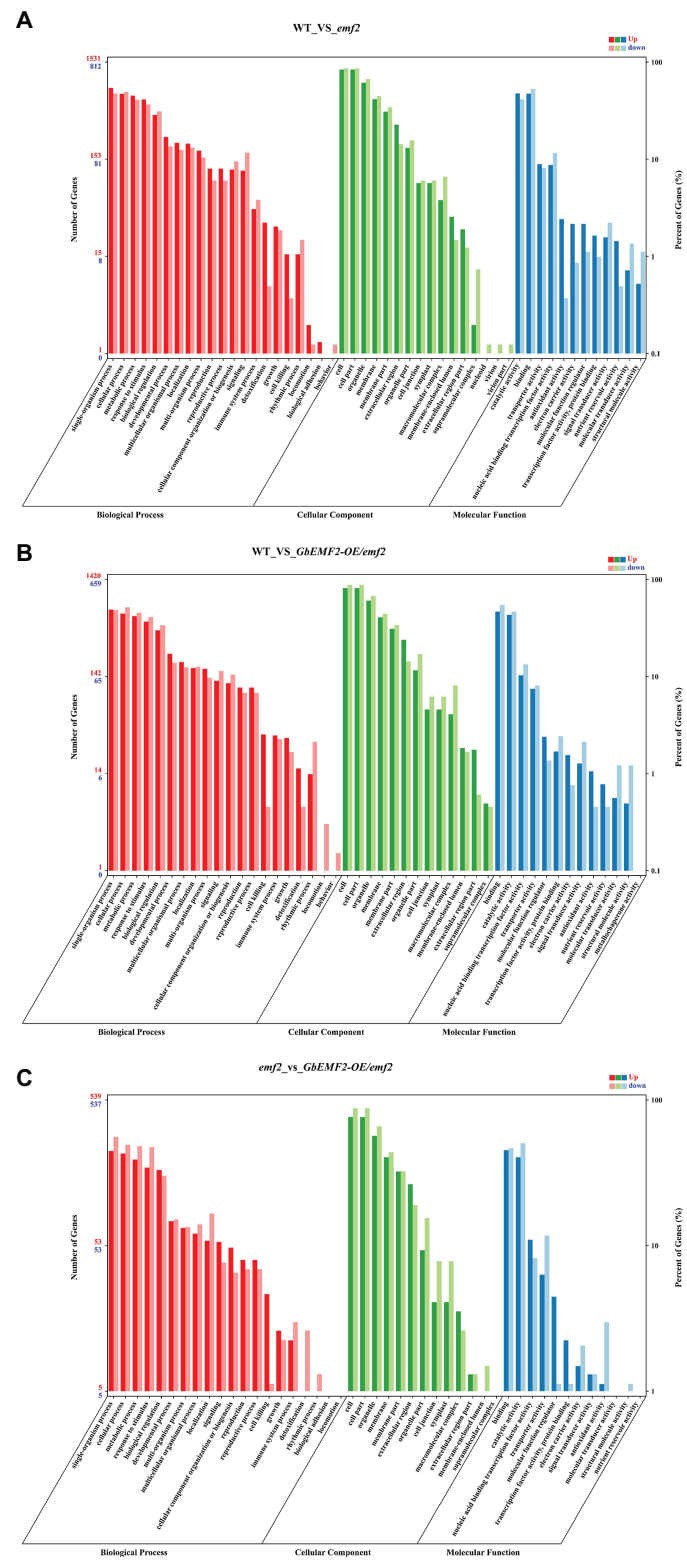
Gene ontology (GO) of differentially expressed genes (DEGs) analysis of WT, *emf2* mutant, and *GbEMF2-OE/emf2* plant. A total of 3,414 DEGs were annotated by at least one of the three categories: biological process, cellular component, and molecular function. **(A)** The GO classification of WT___vs___*GbEMF2-OE*/*emf2*. **(B)** The GO classification of WT___vs___e*mf2*. **(C)** The GO classification of e*mf2* vs___*GbEMF2-OE*/*emf2*. The *x*-axis indicates the level 2 GO terms, and the double *y*-axis means the number of genes and percentage. Red, green, and blue represent upregulated of biological process, cellular component, and molecular function, respectively. Dark red, dark green, and dark blue represent downregulated of biological process, cellular component, and molecular function, respectively.

**Figure 6:**
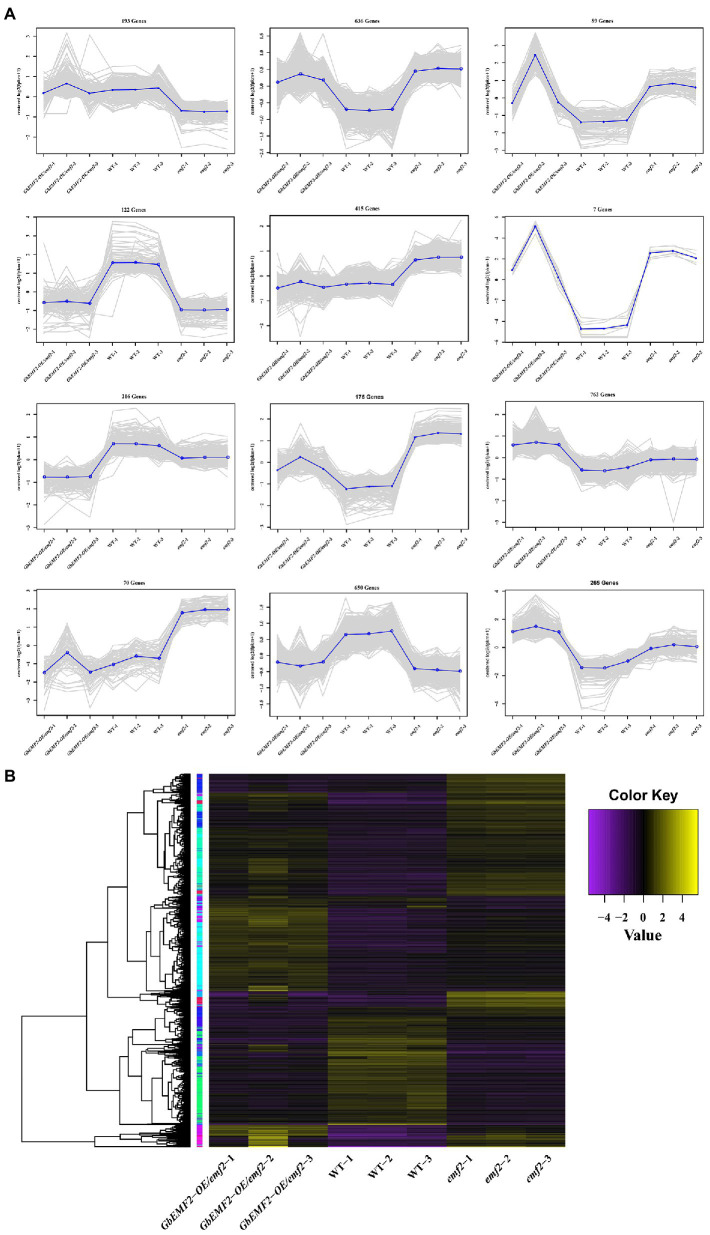
Expression profiles of significant DEGs. **(A)** Clusters of DEGs were obtained by K-means. DEGs were divided into 12 subclasses. **(B)** Heat map for cluster analysis of DEGs by hierarchical cluster combined with K-means method. The change in the expression level is represented by the change in color; purple indicates a lower expression level, and yellow indicates a higher expression level.

To determine the molecular mechanism of early-flowering phenotypes caused by the overexpression of *GbEMF2*, we studied the global expression pattern of *GbEMF2* transgenic *Arabidopsis* plants (*GbEMF2-OE*/*emf2*), *emf2* mutants, and WT plants (CK) using RNA-seq assay. A total of nine transcriptomes were generated from the shoot of WT plants (CK group: WT-1, WT-2, and WT-3), *emf2* mutant (emf2 group: *emf2*-1, *emf2*-2, and *emf2*-3), and GbEMF2 transgenic *Arabidopsis* plants (*GbEMF2* transgenic group: *GbEMF2-OE*/*emf2*-1, *GbEMF2-OE*/*emf2*-2, and *GbEMF2-OE*/*emf2*-3), each group contained three biological replicates and 10 plants per replicate. Clean reads at 97.69–98.03% of each sample could be matched to the reference *Arabidopsis* genome (*A. thaliana*, TAIR10.37). DEGs were first identified through comparisons of the Fragments Per Kilobase of transcript per Million mapped reads (FPKM) values for each gene of the CK group, *emf2* group, and *GbEMF2* group. A total of 3,600 DEGs were detected in three group pairwise comparisons: 2,480 DEGs in WT___vs___*emf2*, 2,186 DEGs in WT___vs___*GbEMF2-OE*/*emf2*, and 1,142 DEGs in *emf2*___vs___*GbEMF2-OE*/*emf2* (Supplementary Figure S4). The GO annotation was conducted to determine the function of the three groups in each comparison. Among these DEGs, 3,413 DEGs were annotated into the GO database that could be classified into three GO categories: biological process, cellular component, and molecular function. Within the biological process category, the most highly represented terms were “single-organism process,” “cellular process,” “metabolic process,” and “response to stimulus.” Within the cellular component category were “cell,” “cell part,” “organelle,” and “membrane.” Within the molecular function category, “catalytic activity” and “binding” were the two most abundant terms (Figures 5A–C; Supplementary Tables S4–S6). In addition, we conducted common expression patterns based on the FPKM of 3,600 DEGs in each sample by using the Euclidean distance algorithm combined with the K-means algorithm. These DEGs were classified into 12 clusters (Figure 6A), which had similar expression patterns in the same subclass (Figure 6B).

**Figure 7:**
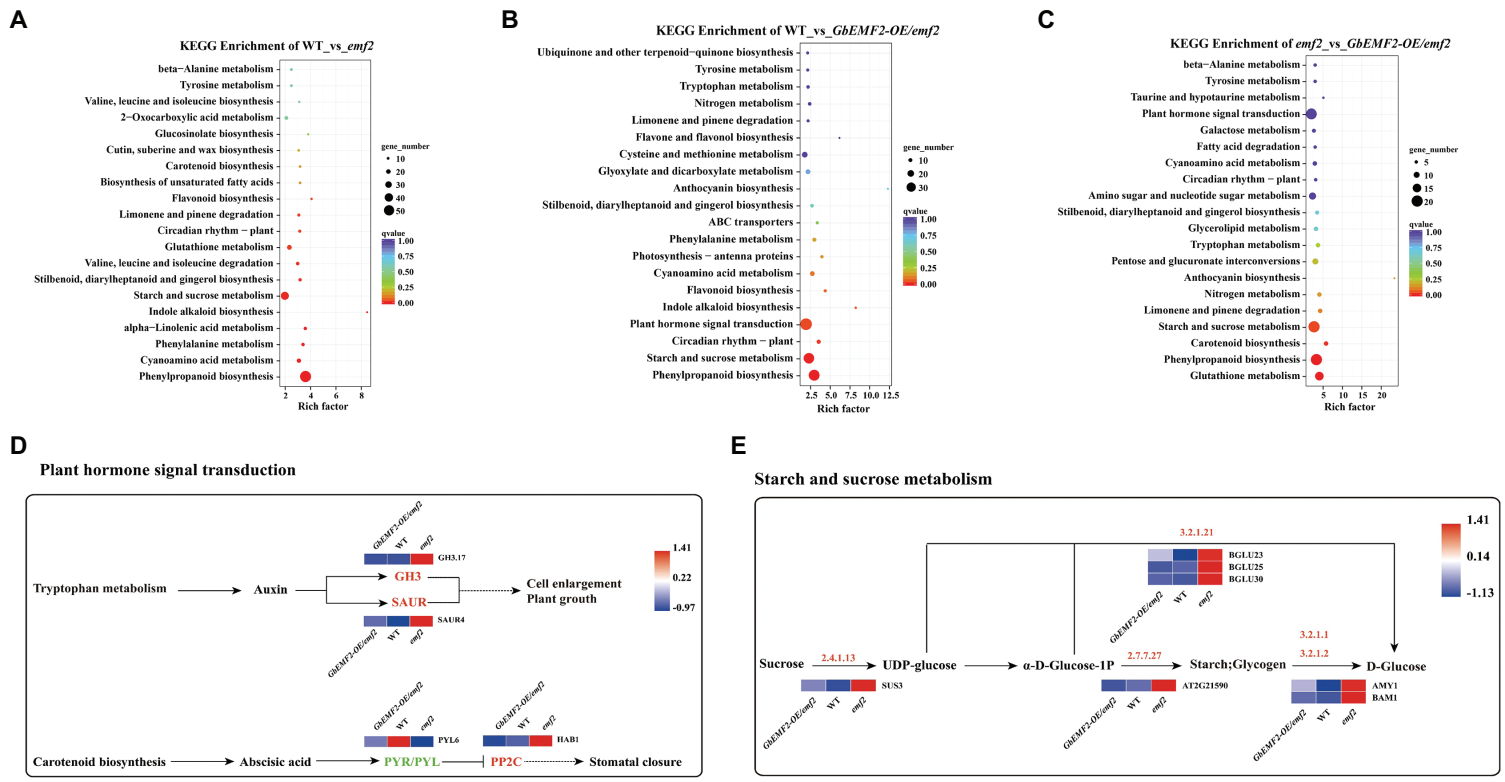
Analysis of DEGs of Kyoto Encyclopedia of Genes and Genomes (KEGG) pathway. **(A)** Analysis of DEGs of top 20 KEGG pathway in WT___vs___e*mf2*. **(B)** Analysis of DEGs of top 20 KEGG pathway in WT___vs___*GbEMF2-OE*/*emf2*. **(C)** Analysis of DEGs of top 20 KEGG pathway in e*mf2*___vs___*GbEMF2-OE*/*emf2*. **(D)** The KEGG metabolic pathway of plant hormone signal transduction. Red indicates that the Fragments Per Kilobase of transcript per Million mapped reads (FPKM) value of genes is high, and blue indicates that the FPKM value of genes is low screened from KEGG orthology (KO) database. **(E)** The KEGG metabolic pathway of starch and sucrose. Red indicates that the FPKM value of genes is highly, and blue indicates that the FPKM value of genes is low screened from KO database.

To further determine how the *EMF2* gene affected metabolic pathways in the flowering process, we predicted the KEGG pathways. In WT___vs___*emf2*, WT___vs___*GbEMF2-OE*/*emf2*, and *emf2*___vs___*GbEMF2-OE*/*emf2* comparisons, 468, 386, and 203 DEGs, respectively, were mapped to 106, 105, and 83 KEGG pathways, respectively (Supplementary Tables S7–S9). The top 20 pathways with significant enrichment were displayed in Figures 7A–C. The DEGs were focused on flowering-related pathways, such as “plant hormone signal transduction (ko04075),” “starch and sucrose metabolism (ko00500),” and “circadian rhythm-plant (ko04712).” Furthermore, the DEGs were focused on other pathways, such as “phenylpropanoid biosynthesis (ko00940)” and “Cyanoamino acid metabolism (ko00460).” Further analysis of the plant hormone signal transduction, four pathways revealed four DEGs, namely, *GH3.17*, *SAUR4*, *PP2C*, and *PYL6* (Figure 7D). In addition, we found seven DEGs, including *BGLU23*, *BGLU25*, *BGLU30*, *Sucrose synthase 3* (*SUS3*), *AT2G21590*, *BAM1*, and *Alpha-amylase 1* (*AMY1*) in starch and sucrose metabolism (Figure 7E).

The transformation of *GbEMF2* resulted in a significant change in most flowering-related DEGs, which might lead to later flowering in transgenic *Arabidopsis*. We screened 40 flowering-related DEGs in Table 1, and these genes were divided into seven categories: flower organ development, ATP-binding cassette (ABC) transporter, auxin pathway, ethylene pathway, abscisic acid pathway, cytochrome P450, and transcription factor. In the *emf2* group, the expression of DEGs was significantly upregulated or downregulated to ultimately influencing flowering. The expression of *FLP2*, *M17*, *LEA2*, *SRF*, *FLC*, *MAF5*, and *MAF5-1*, was not significantly upregulated or downregulated in WT___vs___*GbEMF2-OE*/*emf2*. However, the expression of *FLP2*, *M17*, *LEA2*, and *MAF5* was significantly upregulated in WT___vs___e*mf2* and downregulated in e*mf2*___vs___*GbEMF2-OE*/*emf2*. *FLC* was significantly downregulated in WT___vs___e*mf2* and upregulated in e*mf2*___vs___*GbEMF2-OE*/*emf2*. Two ABC transporters were significantly upregulated in WT___vs___*GbEMF2-OE*/*emf2* and WT___vs___e*mf2*, and downregulated in e*mf2*___vs___*GbEMF2-OE*/*emf2*. In the auxin pathway, SAUR41 and NRT1 were significantly upregulated in WT___vs___e*mf2* and downregulated in e*mf2*___vs___*GbEMF2-OE*/*emf2*, whereas SAUR41 showed no difference in WT___vs___*GbEMF2-OE*/*emf2*. In the abscisic acid pathway, two genes exhibited no difference in WT___vs___*GbEMF2-OE*/*emf2*, but were significantly upregulated in WT___vs___e*mf2* and downregulated in e*mf2*___vs___*GbEMF2-OE*/*emf2*. In the *CYP450* family, six genes were significantly upregulated and downregulated in WT___vs___e*mf2* and e*mf2*___vs___*GbEMF2-OE*/*emf2*, respectively. However, three genes among six genes were significantly upregulated, and other three genes have no difference in WT___vs___*GbEMF2-OE*/*emf2*. In addition, 13 transcription factors had the same expression level in WT___vs___*GbEMF2-OE*/*emf2* but differently upregulated or downregulated in WT___vs___e*mf2* and e*mf2*___vs___*GbEMF2-OE*/*emf2*.

**Table 1:** List of differentially expressed genes associated with flowering.

Category	Gene name	Gene ID	WT_vs_*GbEMF2-OE/emf2*	WT_vs_*emf2*	*emf2*_vs_*GbEMF2-OE/emf2*	Gene description
			Normal/Up/Down	Normal/Up/Down	Normal/Up/Down	
Flower organ development	FLP2	AT5G10625	Normal	Up	Down	Flowering-promoting factor 1-like protein 2
	ANR1	AT2G14210	Up	Up	Down	MADS-box transcription factor ANR1
	FLC	AT5G10140	Normal	Down	Up	MADS-box protein FLOWERING LOCUS C
	MAF5	AT5G65070	Normal	Down	Up	Protein MADS AFFECTING FLOWERING 5
	MAF5-1	AT5G65080	Normal	Down	Up	Protein MADS AFFECTING FLOWERING 5
	APRR3	AT5G60100	Normal	Down	Up	Two-component response regulator-like APRR3
Auxin pathway	SAUR41	AT1G16510	Normal	Up	Down	Auxin-responsive protein SAUR41
Ethylene pathway	ACS2	AT1G01480	Up	Up	Down	1-aminocyclopropane-1-carboxylate synthase 2
	ERF094	AT1G06160	Normal	Down	Up	Ethylene-responsive transcription factor ERF094
	ERF113	AT5G13330	Normal	Up	Down	Ethylene-responsive transcription factor ERF113
	RAP2-6	AT1G43160	Normal	Up	Down	Ethylene-responsive transcription factor RAP2-6
ABC transporter	ABCC10	AT3G59140	Normal	Up	Down	ABC transporter C family member 10
	ABCF2	AT5G09930	Normal	Up	Down	ABC transporter F family member 2
Abscisic acid pathway	CYP707A3	AT5G45340	Normal	Down	Up	Abscisic acid 8&apos;-hydroxylase 3
	PYL6	AT2G40330	Down	Down	Up	Abscisic acid receptor PYL6
	M17	AT2G41260	Normal	Up	Down	Late embryogenesis abundant protein M17
	LEA2	AT1G02820	Normal	Up	Down	Late embryogenis abundant protein 2
Cytochrome P450	CYP71B12	AT5G25130	Up	Up	Down	Cytochrome P450 71B12
	CYP71B26	AT3G26290	Up	Up	Down	Cytochrome P450 71B26
	CYP72A14	AT3G14680	Up	Up	Down	Cytochrome P450 72A14
	CYP72A15	AT3G14690	Normal	Up	Down	Cytochrome P450 72A15
	CYP81F1	AT4G37430	Normal	Up	Down	Cytochrome P450 81F1
	CYP81F4	AT4G37410	Normal	Up	Down	Cytochrome P450 81F4
Transcription factor	WD40	AT4G01870	Up	Up	Down	WD40-like
	NAC003	AT1G02220	Normal	Up	Down	NAC domain-containing protein 3
	NAC047	AT3G04070	Up	Up	Down	NAC transcription factor 47
	NAC090	AT5G22380	Normal	Down	Up	NAC domain-containing protein 90
	WRKY75	AT5G13080	Up	Up	Down	Probable WRKY transcription factor 75
	ATL75	AT1G49200	Down	Down	Up	RING-H2 finger protein ATL75
	BHLH167	AT1G10585	Normal	Up	Down	Transcription factor Bhlh167
	BHLH47	AT3G47640	Normal	Up	Down	Transcription factor bHLH47
	BHLH101	AT5G04150	Normal	Up	Down	Transcription factor bHLH101
	BOA	AT5G59570	Normal	Down	Up	Transcription factor BOA
	HRS1	AT1G13300	Normal	Up	Down	Transcription factor HRS1
	MYB2	AT1G48000	Normal	Up	Down	Transcription factor MYB2
	MYB75	AT1G56650	Normal	Up	Down	Transcription factor MYB75
	SAP12	AT3G28210	Normal	Up	Down	Zinc finger AN1 domain
	AZF1	AT5G67450	Normal	Down	Up	Zinc finger protein AZF1
	BTS	AT3G18290	Normal	Up	Down	Zinc finger protein BRUTUS
	AT1G74770	AT1G74770	Normal	Up	Down	Zinc finger protein BRUTUS-like At1g74770

**Figure 8:**
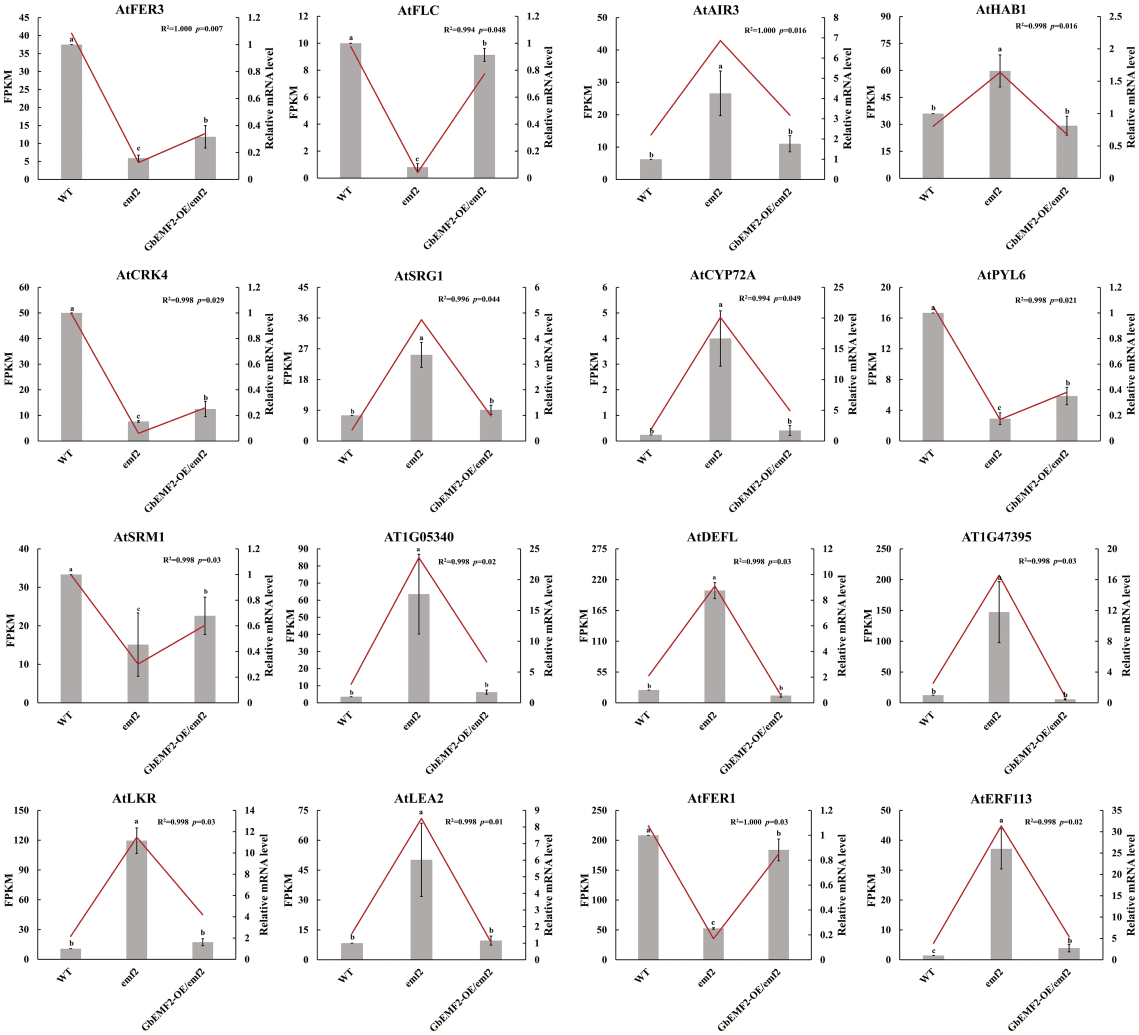
The qRT-PCR validation of the expression patterns of 16 DEGs. Data are mean ± SD of three biological replicates with 10 plants per replicate. Line and block represent RNA-seq and qRT-PCR data. Letters mean significant difference at *p* ≤ 0.05 by the Duncan’s multiple range tests.

The expression patterns for *AtFER3*, *AtFLC*, *AtPYL6*, and *AtFER1* were significantly upregulated in *GbEMF2-OE*/*emf2* compared with the *emf2* group and were significantly upregulated in the WT compared to the *GbEMF2-OE*/*emf2* group. In addition, the expression patterns for *AtLEA2* and *AtERF113* were significantly downregulated in *GbEMF2-OE*/*emf2* compared with the *emf2* group. The expression pattern for *AtERF113* was significantly downregulated in the WT compared with *GbEMF2-OE/emf2* plants. However, relating to the *GbEMF2-OE/emf2* plants, the expression of *AtLEA2* in WT plants showed no difference. To further validate the RNA-seq results, we randomly selected 16 DEGs for the qRT-PCR-based expression analysis in WT plants, *emf2* mutant, and *GbEMF2-OE/emf2* plants (Figure 8). The qRT-PCR data were consistent with the RNA-seq data, which confirmed the accuracy of our transcriptomic analysis. These results showed that *GbEMF2* played a similar role with *AtEMF2*.

## Discussion

The *EMF* gene plays an important role in maintaining vegetative development and repressing flower development. Together, EMF2 protein shares the VEFS-Box domain with homologous FIS2 and VRN (Yoshida et al., 2001). In this study, we characterized a *G. biloba EMBRYONIC FLOWER* gene named *GbEMF2* that contains a VEFS-Box domain (Figure 1) and belongs to one member of the *EMF2* family based on phylogenetic analysis.

### *GbEMF2* Was Predominately Expressed in Reproductive Organs

The expression patterns of *AtEMF2*, *BoEMF2*, and *DlEMF2* and the expression pattern in *G. biloba* orthologue *GbEMF2* had similarities and differences. The *AtEMF2* gene was expressed in developing embryos, vegetative and reproductive shoot meristems, and lateral organ primordia in *A. thaliana* (Yoshida et al., 2001). A similar expression pattern can be found in broccoli, in which the expression of flower buds was the highest, followed by leaves, stems and siliques, and roots (Liu et al., 2012). The *OsEMF2* gene expression level was the highest in the shoot apical meristem and inflorescence meristem, followed by leaves, roots, immature seeds, and calli in rice (Conrad et al., 2014). Xu et al. (2010) also found that the *DlEMF2* gene also was expressed in all bamboo organs. However, the expression level in the shoot organ was higher than that in the inflorescences. In this study, we found that *GbEMF2* was expressed in the roots, stems, leaves, microstrobilus, ovulate strobilus, and young fruits of *G. biloba*. Meanwhile, the expression level in microstrobilus, ovulate strobilus, and young fruits was higher than in roots, stems, and leaves. *GbEMF2* was predominantly expressed in reproductive organs, indicating that *GbEMF2* may be involved in flowering regulation in *G. biloba*.

### Ectopic Expression of *GbEMF2* Led to Delayed Flowering in Transgenic *Arabidopsis*

To further clarify the biological function of *GbEMF2*, we expressed it in *Arabidopsis* driven by the 35S promoter. The flowering time of *GbEMF2-OE/emf2* was later than that of the *emf2* mutant; however, it was earlier than that of the WT. *GbEMF2* restored the phenotype of the *emf2* mutant. This result was consistent with the study in broccoli (Liu et al., 2012). The *emf2* mutants showed an early flowering phenotype that skips rosette growth and directly produces small inflorescences (Yoshida et al., 2001). The expression of *BoEMF2.1* in the *Arabidopsis emf2* mutant partially rescued the mutant phenotype by delaying flowering time and increasing the number of rosette leaves. The *BoEMF2.1* reduced the expression of flower organ identity genes and changed the flowering time gene in *emf2* mutants (Liu et al., 2012). Therefore, *GbEMF2-OE/emf2* experiments verified that *GbEMF2* had the function of repressing plant flowering.

### *GbEMF2* Regulating Flowering-Related DEGs Could Result in Later Flowering in *GbEMF2* Transgenic *Arabidopsis*

**Figure 9:**
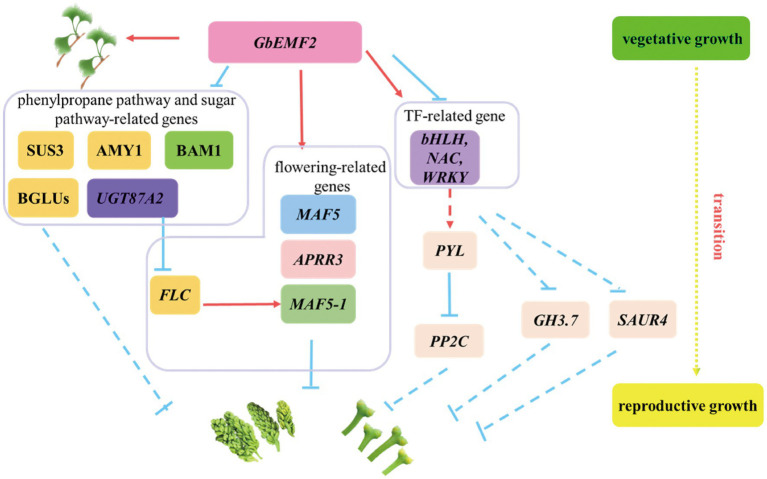
A hypothetical model for the *GbEMF2* regulated other genes controlling flowering in *G. biloba*. Red arrow represents promoting. The inhibition is represented by the blue inverted T line. The solid lines represent by verified in the literature, and the dashed lines represent speculations.

The *MADS-box* gene family has been widely characterized in many woody plants, including *Gossypium hirsutum* (Nardeli et al., 2018), *Populus trichocarpa* (Leseberg et al., 2006), and *G. biloba* (Yang et al., 2016). *MADS* genes function in floral development and regulate flowering time. For example, the *FLC* gene represses the floral integrator genes *SOC1* and *FT* to control flowering (Helliwell et al., 2006). In addition, *MAF* represses flowering by directly activating *TEM1* (Huang et al., 2019). In the present study, *FLC*, *MAF5*, and *MAF5-1* were downregulated and upregulated in WT___vs___*emf2* and *emf2*___vs___*GbEMF2-OE/emf2*. *ANR1* was upregulated and downregulated in WT___vs___*emf2* and *emf2*___vs___*GbEMF2-OE/emf2*. These results indicated that these genes might be regulated by *GbEMF2*, resulting in affecting flower development. We proposed a hypothetical network model for the *GbEMF2* gene by regulating *FLC*, *MAF5*, *ANR1*, and *MAF5-1* to control flowering (Figure 9).

### *GbEMF2* Regulating Phenylpropane Pathway and Sugar Pathway-Related DEGs Could Result in Later Flowering in *GbEMF2* Transgenic *Arabidopsis*

The phenylpropane biosynthesis pathway and sugar pathway were very important secondary metabolic pathways in plants. For example, downregulation of genes in phenylpropane pathway and sugar pathway may ultimately stunt isonuclear alloplasmic male sterility in wheat (Liu et al., 2020). Cinnamoyl-CoA reductase 2 (*CCR2*), β-glucosidase genes, and peroxidase genes were involved in phenylpropane biosynthesis pathway (Cheng et al., 2012; Li et al., 2018). Moreover, pollination changes the expression of β-glucosidase gene 15 (*BGLU15*) and peroxidase gene 47 (*PER47*) to improve the quality of the pears (Li et al., 2018). In our study, we found GbEMF2 changed the expression of *CCR2*, *BGLU23*, *BGLU25*, *BGLU30*, *PER47*, and *PER70* to regulate flower. Furthermore, *BGLU23*, *BGLU25*, and *BGLU30* were annotated sugar pathway. Sugar functions as a source of energy and florigenic signal in plants. The flower induction was mediated by sugar and hormone pathways in apples (Xing et al., 2015). The transcript levels of the *SUS3* gene were the lowest in *Arabidopsis* flower and the highest in siliques (Fallahi et al., 2008). In our study, we found that the transcript levels of the *AtSUS3* gene were the lowest in the WT plant, and highest in the *emf2* mutant; these levels were intermediate in *GbEMF2-OE/emf2* plants. Therefore, we speculated that GbEMF2 controlled flowering by regulating the expression of the *SUS3* gene. *BAM1* and *BAM2* genes are involved in cell division and the differentiation of flowering development (Hord et al., 2006). Additionally, *CIK* genes interact with *BAM1/2* genes and *RPK2* genes to regulate flowering development in *Arabidopsis* (Cui et al., 2018). The *AMY1* gene is not only involved in starch metabolism but also suppresses the expression of *CO* and *FT*. Meanwhile, the expression of the *AMY1* gene was induced by GA and ABA (Jie et al., 2009). The *BAM1* and *AMY1* genes were significantly expressed in e*mf2*___vs___*GbEMF2-OE*/*emf2*. Therefore, the two genes may be regulated by the *GbEMF2* gene. Given the joint analysis of our data and flowering-related genes, we speculated that *GbEMF2* may control the flowering process by regulating the expression of *SUS3*, *BAM1*, and *AMY1* (Figure 9).

### *GbEMF2* Regulating TF-Related DEGs Could Result in Later Flowering in *GbEMF2* Transgenic *Arabidopsis*

The TFs, including *MYB*, *bHLH*, *NAC*, and *WRKY*, play essential roles in the reproductive development of plants. The *MYB* genes are potentially involved in flower development in *Rafflesia cantleyi* (Amini et al., 2019). Some genes of *bHLH* families act as flower developmental regulators that control flowering time (Ito et al., 2012). In our study, *AtbHLH47*, *AtbHLH101*, and *AtbHLH167* were upregulated in WT___vs___*emf2* and downregulated in *emf2*___vs___*GbEMF2-OE/emf2*; no significant difference was found in WT___vs___*GbEMF2-OE/emf2* (Table 1), which showed that *GbEMF2* might regulate *bHLH* genes to control flower. Moreover, losing NAC transcription factors, including *ANAC050*, *ANAC052*, and *ANAC075*, led to early flowering phenotype (Fujiwara and Mitsuda, 2016). In this study, the expression of *NAC003* changed significantly in three groups. The expression of *NAC047* exhibited no significant difference in WT___vs___*GbEMF2-OE/emf2*, but showed a significant difference between *emf2* and *GbEMF2-OE/emf2*, which implied that *GbEMF2* regulated flower development by influencing *NAC*. In addition, *WRKY* regulates flowering time in different ways. For instance, an opposite changing trend was exhibited between *WRKY12* and *WRKY13*. The *WRKY12* positively regulates flowering time under short-day, whereas *WRKY13* serves as a negative regulator (Li et al., 2016b). Thus, we speculated that *GbEMF2* might regulate flowering by *MYB*, *bHLH*, *NAC*, and *WRKY*. Overall, based on these results, a hypothetical network model was proposed for the *GbEMF2* gene to regulate *TFs* further influencing flowering (Figure 9).

## Conclusion

In summary, the present study cloned and characterized *GbEMF2* from *G. biloba*. The GbEMF2 protein contains a conserved VEFS-Box domain, which was homologous with VRN2 and FIS2 proteins. The expression level of *GbEMF2* in reproductive organs was significantly higher than that in vegetative organs. Overexpressing the *GbEMF2* in transgenic *Arabidopsis* plants, the flowering time of ectopic was later than that of *emf2* mutant plants but was earlier than that of the WT plant. In addition, *GbEMF2* overexpression in transgenic plants changed the expression levels of flowering-related DEGs, sugar-related DEGs, and TF-related DEGs. Based on our results, we speculated that GbEMF2 may regulate the flowering of *G. biloba* by regulating *MADS* genes, *BAM1*, *AMY1*, or interacting with NAC, BHLH, and WRKY transcription factors. Our study provides a foundation for understanding *GbEMF2*, which is involved in the flowering of *G. biloba*.

## Data Availability Statement

The raw sequence data reported in this paper have been deposited in the Genome Sequence Archive (Wang et al., 2017) in National Genomics Data Center (CNCB-NGDC Members and Partners, 2021), China National Center for Bioinformation/Beijing Institute of Genomics, Chinese Academy of Sciences, under accession number CRA003802 that are publicly accessible at https://bigd.big.ac.cn.

## Author Contributions

FX designed the whole experiment and drafted the manuscript. XZ performed the part experiment and wrote the manuscript. JYa contributed to cDNA cloning and qRT-PCR analysis. LW and XZ performed the transgenic experiment. XL, WZ, and YL guided the experiment. JYe, SC, and GW revised the manuscript. All authors contributed to the article and approved the submitted version.

### Conflict of Interest

The authors declare that the research was conducted in the absence of any commercial or financial relationships that could be construed as a potential conflict of interest.

## Footnotes

^1^ https://sg.idtdna.com/PrimerQuest/Home/Index

^2^ https://blast.ncbi.nlm.nih.gov/

^3^ https://www.lynnon.com/dnaman.html

^4^ http://downloads.fyxm.net/Clustal-X-58923.html

^5^ https://www.megasoftware.net/

^6^ https://ccb.jhu.edu/software/stringtie/index.shtml

^7^ ftp://ftp.ncbi.nih.gov/blast/db/

^8^ http://www.uniprot.org/

^9^ http://www.geneontology.org/

^10^ http://www.ncbi.nlm.nih.gov/COG/

^11^ http://www.ncbi.nlm.nih.gov/KOG/

^12^ http://pfam.xfam.org/

^13^ http://www.genome.jp/kegg/

## Supplementary Material

Supplementary Figure S1 Nucleotide sequence and deduced amino acid sequence of GbEMF2: the red box is the start and stop codons, and the primer sequences were underlined.

Supplementary Figure S2 Screening of *GbEMF2* transgenic *Arabidopsi*s plants with T_1_ generation.

Supplementary Figure S3 The PCR detection of GbEMF2 transgenic *Arabidopsis* plants. **(A)** Screening of GbEMF2 transgenic *Arabidopsis* plants with T_2_ generation. Black arrows indicate nontransgenic plants. GbEMF2-1, GbEMF2-2, and GbEMF2-3 are three strains of transgenic *Arabidopsis*. **(B)** GUS staining of GbEMF2 transgenic *Arabidopsis* plants. **(C)** Extraction of DNA from *Arabidopsis* plants. 1: *Arabidopsis emf2* mutant plants; 2: *GbEMF2-OE/emf2*-1 strain; 3: *GbEMF2-OE/emf2*-2 strain; and 4: *GbEMF2-OE/emf2*-3 strain. **(D)** Cloning of the *GbEMF2* gene. M: 5000 Marker; 1–5: GbEMF2 Transgenic *Arabidopsis* plants; 6–8: *Arabidopsis emf2* mutant plants.

Supplementary Figure S4 Venn plot of WT, *emf2* mutant, and *GbEMF2-OE/emf2* plant comparison. G0: WT___vs___*GbEMF2-OE*/*emf2*; G1: WT___vs___e*mf2*; G2: e*mf2*___vs___*GbEMF2-OE*/*emf2*.

Supplementary Table S1 Primer sequence used in this study.

Supplementary Table S2 Similarity of GbEMF2 protein and other plant EMF2 protein sequences.

Supplementary Table S3 Su(Z)12 homologous protein sequences used in the phylogenetic analysis.

Supplementary Table S4 Gene ontology analysis data in WT___vs___e*mf2*.

Supplementary Table S5 Gene ontology analysis data in WT___vs___*GbEMF2-OE*/*emf2*.

Supplementary Table S6 Gene ontology analysis data in e*mf2*___vs___*GbEMF2-OE*/*emf2*.

Supplementary Table S7 Kyoto Encyclopedia of Genes and Genomes analysis data in WT___vs___e*mf2*.

Supplementary Table S8 Kyoto Encyclopedia of Genes and Genomes analysis data in WT___vs___*GbEMF2-OE*/*emf2*.

Supplementary Table S9 Kyoto Encyclopedia of Genes and Genomes analysis data in e*mf2*___vs___*GbEMF2-OE*/*emf2*.
