# Supplementary figures and images for: Functional Characterization of the EMBRYONIC FLOWER 2 Gene Involved in Flowering in Ginkgo biloba
Source: Front Plant Sci. 2021 Jun 21;12:681166. doi: 10.3389/fpls.2021.681166 (PMC8451716; doi:10.3389/fpls.2021.681166)

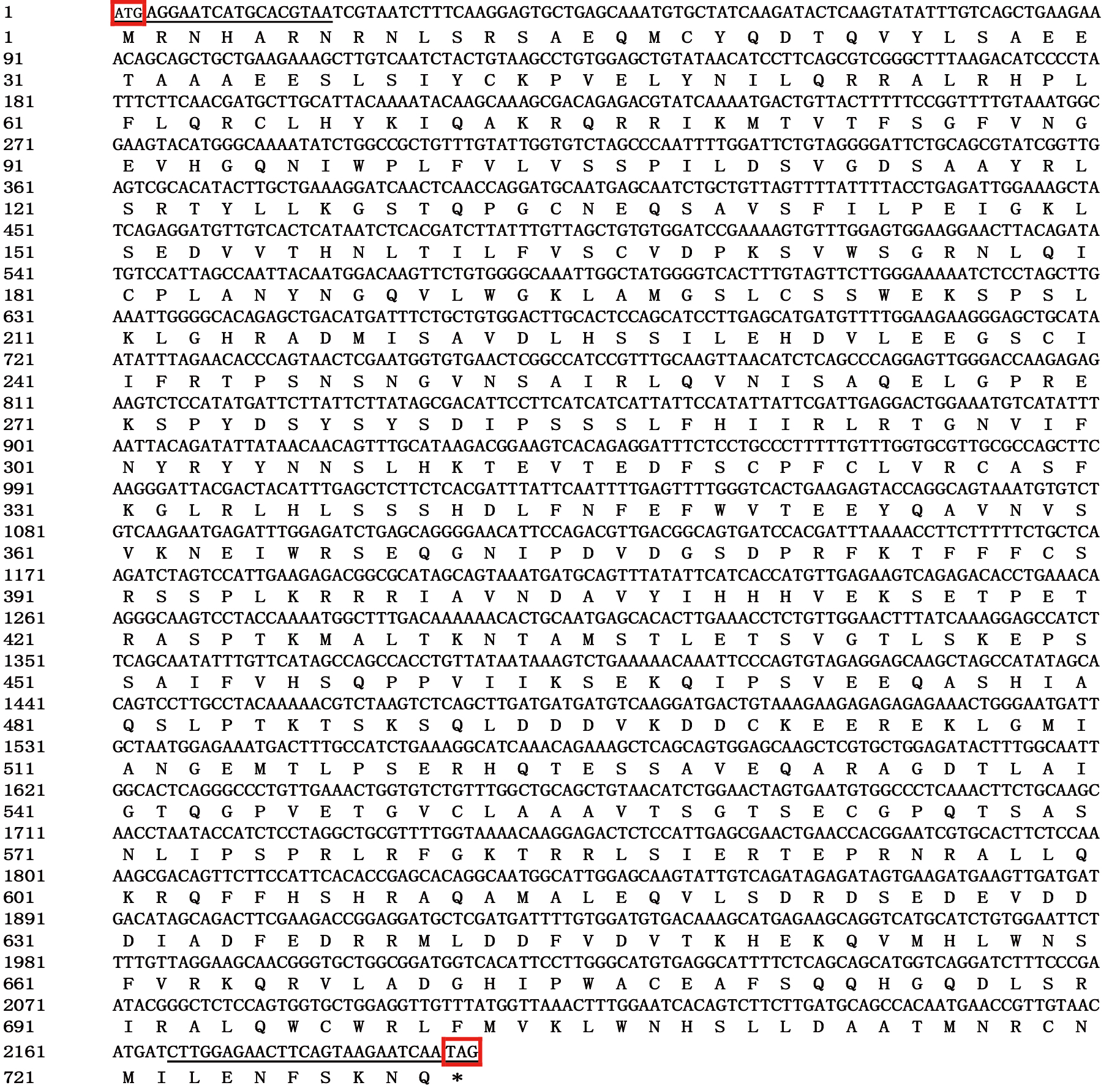

Supplement: Supplementary file 1 [file Data_Sheet_1.zip › Figure S1.JPEG]

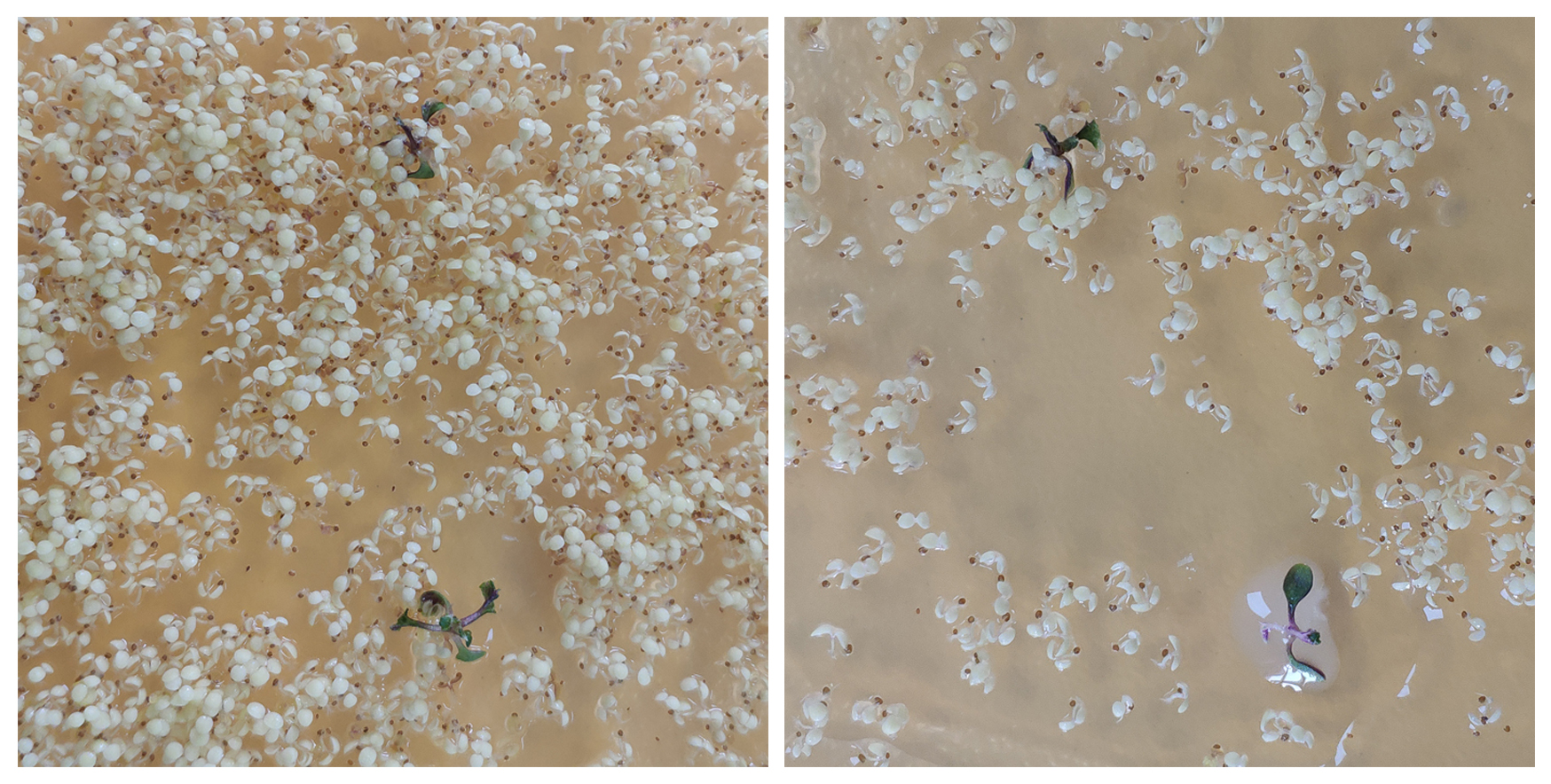

Supplement: Supplementary file 1 [file Data_Sheet_1.zip › Figure S2.JPEG]

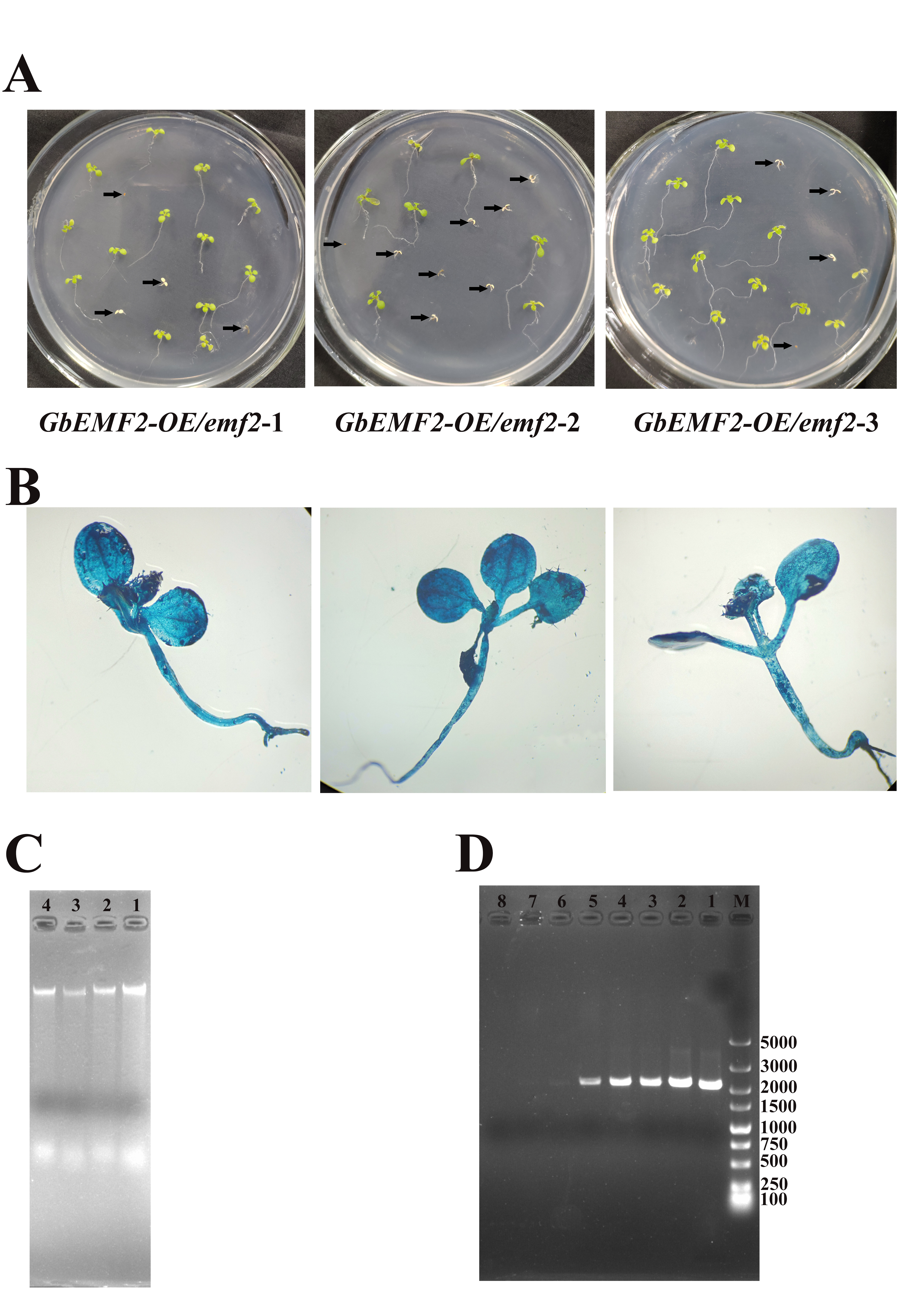

Supplement: Supplementary file 1 [file Data_Sheet_1.zip › Figure S3.JPEG]

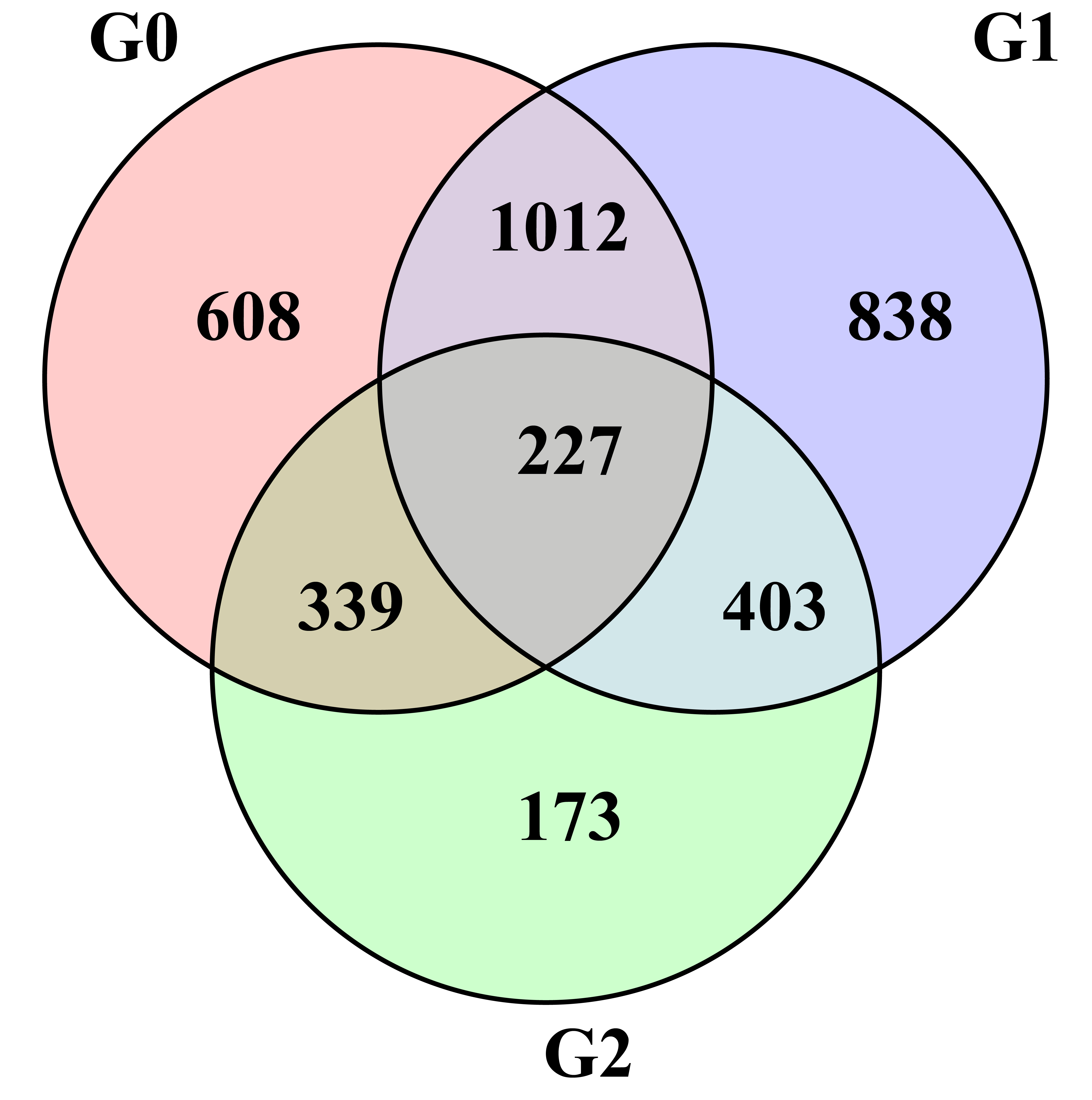

Supplement: Supplementary file 1 [file Data_Sheet_1.zip › Figure S4.JPEG]
